# Lactic Acid Bacteria as Biopreservation Against Spoilage Molds in Dairy Products – A Review

**DOI:** 10.3389/fmicb.2021.819684

**Published:** 2022-01-26

**Authors:** Ce Shi, Maryam Maktabdar

**Affiliations:** Section of Food Microbiology and Fermentation, Department of Food Science, Faculty of Science, University of Copenhagen, Copenhagen, Denmark

**Keywords:** lactic acid bacteria, mold spoilage, dairy products, biopreservation, antifungal metabolites, manganese depletion

## Abstract

Mold spoilage of dairy products such as yogurt is a concern in dairy industry. Not only does it lead to substantial food waste, economic losses, and even brand image damage, but it may also cause public health concern due to the potential production of mycotoxin. Good hygiene practices are necessary to prevent contamination, but contamination may nevertheless occur at the production site and, not least, at the site of the consumer. In recent years, there has been a growing interest from consumers for “clean label” food products, which are natural, less-processed, and free of added, chemical preservatives, and a wish for shelf lives of considerable length in order to minimize food waste. This has sparked an interest in using lactic acid bacteria (LAB) or their metabolites as biopreservatives as a way to limit the growth of spoilage organisms in dairy products. A range of compounds produced by LAB with potential antifungal activity have been described as contributing factors to the inhibitory effect of LAB. More recently, growth inhibition effects caused by specific competitive exclusion have been elucidated. It has also become clear that the sensitivity toward both individual antifungal compounds and competition mechanisms differ among molds. In this review, the main spoilage molds encountered in dairy products are introduced, and an overview of the antifungal activity of LAB against different spoilage molds is presented including the main antifungal compounds derived from LAB cultures and the sensitivity of the spoilage molds observed toward these compounds. The recent findings of the role of competitive exclusion with emphasis on manganese depletion and the possible implications of this for biopreservation are described. Finally, some of the knowledge gaps, future challenges, and trends in the application of LAB biopreservation in dairy products are discussed.

## Introduction

Fresh, fermented milk products such as yogurt have been known for milleniums and they are consumed under different names in many parts of the world. Although the fermentation ensures a longer shelf life, spoilage of these products by molds is, nevertheless, a major problem, which not only leads to substantial food waste and global economic losses every year, but may also threaten human health due to the potential production of mycotoxins (Bullerman, 1981). Molds are considered as common contaminants due to being ubiquitous in nature and thriving in a wide range of environmental conditions (Sadiq et al., 2019). Indeed, dairy-associated spoilage molds are highly diverse at the genus and species level. So far, up to 100 mold species have been identified as being responsible for dairy product spoilage, including *Penicillium* and *Mucor* spp. (Garnier et al., 2017a). The presence of spoilage molds in dairy products leads to different types of food spoilage, including visible growth on the surface of products, visible alterations in color and/or texture, and formation of off-odors (Ledenbach and Marshall, 2009). Besides, mycotoxins produced by some molds, such as ochratoxin A, citrinin, and roquefortin C in cheese (Hymery et al., 2014), pose a considerable threat to food safety and human health. Traditional hurdle technologies are implemented to prevent and/or limit fungal growth in dairy products, such as good hygiene practices, a hazard analysis critical control point (HACCP) system, and cleanroom technologies. However, various factors should be taken into account when choosing the most suitable preventive and control methods, such as the impact on the characteristics of food products, consumer perception and acceptance, food storage conditions, cost, etc. In addition, fungal resistance toward chemical preservatives has been reported recently (Crowley et al., 2013; Garnier et al., 2017a). The health concern with food safety issues and demand for natural, less processed, or preservative-free food products from consumers are increasing. In this context, the development of new preservation technologies as alternatives to chemical additives is urgent. In recent years, using microorganisms or their metabolites to control unwanted organisms and extend food shelf-life and increase food safety has gained increasing interest (Yépez et al., 2017; Quattrini et al., 2019).

Currently, biopreservation by lactic acid bacteria (LAB) is the most promising alternative candidates to chemical preservatives in dairy industry due to their Generally Regarded as Safe (GRAS) status and Qualified Presumption of Safety (QPS) status in the United States and EU, respectively. The antifungal activity of LAB against spoilage molds has been studied not only in laboratory media but in a range of fermented food products, such as yogurt, cheese, and bread (Schwenninger and Meile, 2004; Delavenne et al., 2013, 2015; Garnier et al., 2017b; Ouiddir et al., 2019). The potential mechanisms behind the antifungal activity of LAB have mainly been investigated through the characterization and identification of microbial metabolites. The LAB metabolites mainly include organic acids, fatty acids, hydrogen peroxide (H_2_O_2_), proteinaceous compounds, reuterin, and some volatiles, such as diacetyl (Crowley et al., 2013; Aunsbjerg et al., 2015a). The antifungal activities of some microbial metabolites have been determined in fungal growth inhibition (Ebrahimi et al., 2020; Garnier et al., 2020). However, the amount of the metabolites has generally been found in concentrations that are much lower than their minimal inhibitory concentrations (MICs) (Siedler et al., 2019), and it has therefore been suggested that the antifungal metabolites might act in synergy. Noteworthy, the depletion of manganese has been reported to be a novel mechanism in LAB to delay the growth of spoilage fungi in dairy products (Siedler et al., 2020). This finding could be interesting for the dairy industry to prevent and control fungal contamination since manganese depletion is a non-microbicidal effect. A better understanding of the interactions between LAB cultures/metabolites and spoilage molds, and the possible mechanism behind the antifungal activity of the cultures/metabolites will facilitate the development of new bioprotective cultures in food industry.

Overall, the present review aims to introduce the main spoilage molds encountered in dairy products, mainly *Penicillium* and *Mucor* strains, and to give an overview of the inhibitory effects of LAB against different spoilage molds. Concerning the antifungal mechanisms of the LAB bioprotective cultures, the main antifungal compounds derived from LAB cultures and the sensitivity of the spoilage molds observed toward these compounds are summarized. In addition, the role of competitive exclusion with emphasis on manganese depletion and the possible implications of this for biopreservation are described. Finally, some of the knowledge gaps, future challenges, and trends in the application of LAB biopreservation in fresh fermented dairy products are discussed.

## Fungal Spoilage of Dairy Products

### Dairy-Associated Spoilage Molds

In general, dairy-associated spoilage molds can grow well at low temperature, low water activity, low pH, and low oxygen tension atmospheres. Mold contamination of dairy products can be found from the environmental sources including primary production environments such as farms and dairy processing environment (Nicole et al., 2022). In the dairy environment, mold spores become airborne, and contaminate the dairy products after heating. For heat-treated dairy products, the occurrence of mold contamination might be due to the post-processing contamination during packing or the heat-resistance of mold spores (Garnier et al., 2017a). Pitt and Hocking (2009) isolated some heat-resistant species from cream cheese and heat-treated dairy beverage such as *Eupenicillium brefeldianum* and *Talaromyces macrosporus*, respectively. For Ultra-High Temperature (UHT)-treated dairy products, the occurrence of contamination might be due to the post-processing, such as the isolation of *Fusarium oxysporum* from UHT-flavored milk beverages (Pitt and Hocking, 2009).

Most mold species that are responsible for dairy products spoilage belong to the Ascomycota and Mucoromycota phyla, including various *Penicillium, Mucor, Cladosporium, Aspergillus, Fusarium*, and *Geotrichum* spp. (Pitt and Hocking, 2009). Among Ascomycota, all identified spoilage molds belong to the Pezizomycotina subdivision (except *Lecanicillium lecanii*) (Lund et al., 1995), and the Eurotiomycetes class is the most represented; among Eurotiomycetes, *Penicillium* is more frequently reported (around 40 species), followed by *Aspergillus* with 10 species (Garnier et al., 2017a). Among the Mucoromycotina sub-phylum, *Mucor*, *Rhizopus* and *Thamnidium* have already been isolated from contaminated dairy products. Sulaiman et al. (2014) found *Rhizomucor variabilis* in Greek yogurt samples; Snyder et al. (2016) reported the spoilage of yogurt by *Mucor circinelloides*; and Buehler et al. (2017) identified *Mucor*, *Rhizopus* and *Sistotrema* in yogurt. Among these dairy-associated spoilage molds, the dairy contamination by *Penicillium* and *Mucor* are frequently reported.

*Penicillium* spp., one of the main genera involved in dairy product spoilage, are mainly isolated from hard and semi-hard cheeses, butter, yogurt, milk, and goat or ewe milk cheeses (Table 1). It is reported that 50∼90% of spoilage molds in cheese are *Penicillium* species. Among these species, *P. commune, P. nalgiovense*, and *P. roqueforti* play a dominating role in the spoilage microbiota, while other *Penicillium* species appear to be found less often, such as *P. expansum, P. granulatum*, and *P. palitans* (Nicole et al., 2022). Buehler et al. (2017) reported that the most common genus isolated from yogurt, milk, and cheese was *Penicillium*, such as *P. roqueforti*. *P. glabrum* is of common occurrence in raw materials and sometimes causes spoilage of cheese and margarine (Pitt, 2014). In yogurt and packaged Grana cheese, *P. chrysogenum* was identified as the main spoiler (Cappa and Cocconcelli, 2001). The physiology of *Penicillium* species is highly evolved, the growth of *Penicillium* strains has a very low nutritional requirement. This helps adapt to an extensive range of living habitats. *Penicillium* species are capable of growing at low pH (down to pH 3 even pH 2), low temperature (below 5°C, and some at 0°C), low water activity (around 0.8), and some species such as *P. roqueforti* can grow at low oxygen levels (normally 2%, slow growth in 0.5% O_2_). Some *Penicillium* species with ascosporic states display notable heat resistance. A few *Penicillium* species, in particular *P. roqueforti*, exhibit preservative resistance. However, their growth can be inhibited at a high CO_2_ level (40%) (Pitt, 2014).

**TABLE 1:** Diversity of spoilage *Penicillium* strains isolated from contaminated dairy products.

Mold	Dairy products	References
*Penicillium commune*	Yogurt	Shi and Knøchel, 2021
	Cheese	Makki et al., 2020
	Hard Cheese, Yogurt	Garnier et al., 2017b
*Penicillium adametzioides*	Cream	Garnier et al., 2017b
*Penicillium bialowiezense*	Hard Cheese, Yogurt	Garnier et al., 2017b
*Penicillium fellutanum*	Cream Cheese	Garnier et al., 2017b
*Penicillium palitans*	Cream Cheese	Garnier et al., 2017b
*Penicillium spp*	Caprine and Ovine Cheese	Griffin et al., 2020
*Penicillium crustosum*	Yogurt	Shi and Knøchel, 2021
*Penicillium citrinum*	Yogurt	Makki et al., 2020
*Penicillium roqueforti*	Crème Fraiche	Shi and Knøchel, 2021
*Penicillium expansum*	Fresh Cheese	Nguyen Van Long et al., 2017
*Penicillium aurantiogriseum*	Cheddar Cheese	Ahmed et al., 2020
*Penicillium corylophilum*	Cheddar Cheese	Ahmed et al., 2020
*Penicillium implicatum*	Cheddar Cheese	Ahmed et al., 2020
*Penicillium paxilli*	Cheddar Cheese	Ahmed et al., 2020
*Penicillium solitum*	Pecorino Cheese	Agnolucci et al., 2020
	Hard Cheese, Yogurt, and Yogurt drink	Garnier et al., 2017b
*Penicillium discolor*	Pecorino Cheese	Agnolucci et al., 2020
	Semi-soft Cheese	Lund et al., 1995
*Penicillium verrucosum*	Pecorino Cheese	Agnolucci et al., 2020
*Penicillium brevicompactum*	Cheese	Nguyen Van Long et al., 2017
*Penicillium bovifimosum*	Ovine raw milk	Marín et al., 2015

*Mucor* species are found widespread in nature, including soil, air, and raw materials or foodstuffs. To date, approximately 20 *Mucor* species have been isolated from various foods. In food industry, *Mucor* spp. display an ambivalent behavior. On the one hand, *Mucor* spp. are used as an adjunct technological organism to obtain the desired qualities of a final product, such as cheeses (Hermet et al., 2012). On the other hand, *Mucor* spp. can be a contaminant in food products due to their highly saccharolytic and proteolytic capacity (Orlowski, 1991). Dairy products, fresh fruit and vegetables, cereals, nuts, and meat products are regularly contaminated by *Mucor* spp. (Table 2). Five important *Mucor* species are regarded to be related to food spoilage (*M. circinelloides*, *M. hiemalis, M. piriformis, M. plumbeus*, and *M. racemosus*) (Botha and Botes, 2014). The main *Mucor* species encountered in cheeses are *M. circinelloides*, *M. fuscus*, *M. lanceolatus*, *M. plumbeus*, and *M. racemosus* (Morin-Sardin et al., 2016). Garnier et al. (2017a) identified *M. circinelloides*, *M. racemosus*, and *M. spinosus* from hard cheese. In traditional Slovak sheep cheese, *M. circinelloides* was identified as the predominant fungus (Laurenčík et al., 2008). Some fungal genera can produce toxic secondary metabolites, mycotoxins. For example, *M. circinelloides* is considered as an opportunistic pathogen due to the capacity of producing the mycotoxin 3-nitropropionic acid (Hollmann et al., 2008). *Mucor* species are able to aerobically utilize a wide variety of carbon sources, fermenting carbohydrates and ammonia or organic nitrogen. For the growth conditions, *Mucor* species are able to grow at temperatures ranging from 0°C (e.g., *M. flavus*, *M. piriformis*, *M. plasmaticus*, and *M. racemosus*) to 40°C (e.g., *M. recurvus*), most *Mucor* species are able to grow and sporulate at temperatures from 20°C to 30°C; *Mucor* species occur at pH values of between 4 and 8; the limiting water activity value for *Mucor* seems to be between 0.92 and 0.93 (Botha and du Preez, 1999).

**TABLE 2:** Diversity of spoilage *Mucor* strains isolated from contaminated dairy products.

Mold	Dairy products	References
*Mucor spp.*	Caprine and Ovine Cheese	Griffin et al., 2020
*Mucor racemosus*	Yogurt	Makki et al., 2020
	Hard Cheese	Garnier et al., 2017b
	Hard or semi-hard Cheese	Kure and Skaar, 2000
	Cheese	Botha and Botes, 2014
*Mucor genevensis*	Yogurt	Makki et al., 2020
*Mucor lanceolatus*	Cow’s milk	Nguyen Van Long et al., 2017
*Mucor circinelloides*	Yogurt	Shi and Knøchel, 2021
	Yogurt	Lee et al., 2014
	Hard or semi-hard Cheese	Garnier et al., 2017b
	Sheep Cheese	Laurenčík et al., 2008
	Milk powder	Botha and Botes, 2014
	Yogurt	Pitt and Hocking, 2009
*Mucor spinosus*	Hard Cheese	Garnier et al., 2017b
*Mucor hiemalis*	Buffalo, goat, or sheep Cheese, hard or semi-hard Cheese, Yogurt	Pitt and Hocking, 2009
*Mucor plumbeus*	Dairy products	Botha and Botes, 2014
	Hard Cheese	Kure et al., 2001

### Response of Fungi Toward Manganese

Manganese is one of the most important trace element nutrition of fungi (Yogeswari, 1948). The supplementation of manganese gave a better dry weight of fungi after incubation. Likewise, Rawla (1969) also showed that Mn, Fe and Zn were essential for fungal growth whereas the rest of the trace elements were not found essential. The growth of *Nigrospora oryzae* required an optimum concentration of 100 ppm Mn for its growth. Recently, Siedler et al. (2020) reported that the growth of both yeast and molds was associated with Mn, the deficiency of Mn led to the fungal growth inhibition. However, it is worth noting that the concentrations of Mn beyond the optimum level progressively inhibit the fungal growth. Trace element studies were also carried out by Thind and Mandahar (1968) on five pathogenic fungi. Out of the 15 trace elements tested, Mn Fe, Zn, and Cu were found to be essential for the growth of the five fungi. The optimal concentrations in ppm of Mn for these fungi were as follows: *Cercospora hibiscina*, 1 ppm; *Cercospora withaniae*, Mn 10⋅ppm; *Cercospora crotalariae*, 0.1 ppm; *Monochaetia* sp., 0.1 ppm; *Pestalotia theae*, 0.01 ppm. In addition, one recent research revealed that 0.1 mg/L Mn significantly inhibited the mycelial growth of *Phytophthora nicotianae* (Luo et al., 2020). Furthermore, it was observed that molds require manganese for the production of secondary metabolites, e.g., patulin production in *Penicillium urticae* (*P. urticae*) (Scott et al., 1986). The presence of manganese was essential for the conversion of a pathway intermediate (6-methylsalicylic acid) in patulin production in *P. urticae*, and in manganese-deficient media only a little amount of patulin was produced. Similarly, Dombrink-Kurtzman and Blackburn (2005) reported that the supplementation of the culture media with 152 μM of manganese increased the production of mycotoxin (patulin) in a high amount in different species of *Penicillium*. On the other hand, *Penicillium* spp. are described as prominent organisms to remove the metals from nature by active uptake of metal ions, such as industrial waste-water (Singh, 2006). For example, *Penicillium italicum* was reported to be a useful tool for uptake of Mn(II) (Mendil et al., 2008).

## Prevention and Control of Spoilage Fungi in Dairy Preservation

The microbiological quality of dairy products should be controlled from the raw materials to the finished product. In order to reduce fungal contamination in dairy products, both preventive and control approaches are usually used together. Preventive methods indicate the methods that can avoid contamination or during product manufacturing and processing, such as packaging in aseptic conditions, the utilization of air filtration systems, good manufacturing practices, and HACCP system implementation. Good manufacturing and distribution practices (GMDPs), an indispensable part of every food quality system, concludes two essentials: selecting good quality raw materials and preventing cross-contamination, and avoiding or retarding the growth of unwanted organisms (Garnier et al., 2017a). GMDPs rely on the implemented HACCP system, a systematic approach to identify, evaluate, and control hazards that threaten food hygiene (Motarjemi, 2014).

Control methods are defined as the methods that either retard or inhibit the growth of undesired organisms in food products, such as inactivation treatment (heat and high-pressure treatments), temperature control, and chemical control (Garnier et al., 2017a). In addition, biopreservation has gained much attention in recent years. Biopreservation indicates that using natural organisms (cultures) or their microbial metabolites such as weak acids, bacteriocins, and food-grade enzymes to extend food shelf-life and increase food safety (Holzapfel et al., 1995).

## Lactic Acid Bacteria and Their Antifungal Performance

### Lactic Acid Bacteria

Lactic acid bacteria are a group of microorganisms that are aerotolerant, acid-tolerant and catalase-negative (some LAB strains are capable of producing pseudocatalase) but non-sporulating, non-motile (with some exceptions, some *Latilactobacillus curvatus* strains are motile), and non-respiring gram-positive cocci or rods (Cousin et al., 2014; Liu et al., 2014; Mora-Villalobos et al., 2020). LAB, a ubiquitous bacterial group, are found in the environments that rich in available carbohydrate substrates, including the niches of fermented dairy products, meat, vegetable origin, human and animal cavities, such as the gastrointestinal and urogenital tracts (Liu et al., 2014).

Due to the lack of a functional respiratory system, LAB obtain the energy through substrate-level phosphorylation following two basic hexose fermentative pathways: homofermentative and heterofermentative pathways. The homofermentative pathway is based on the Embden-Meyerhof-Parnas pathway, lactic acid is produced virtually as the end-product of the anaerobic metabolism. The heterofermentative pathway is also known as the pentose phosphoketolase pathway or the 6-phosphogluconate pathway. The end-products include not only lactic acid, but also significant amounts of CO_2_ and ethanol or acetate (Liu et al., 2014).

It is well known that the growth and metabolic activity of LAB require an appropriate biochemical and biophysical environment, although it may vary among the species, even among the strains (Hayek and Ibrahim, 2013). In general, biophysical environmental factors include pH, temperature, water activity, and redox potential (Lechiancole et al., 2002). Regarding biochemical conditions, the culture media of LAB usually contain carbohydrates (carbon source), amino acids, peptides, nucleic acid derivatives, fatty acids esters, minerals, and vitamins (Hébert et al., 2009). Minerals play a key role in microbial growth and enzymatic activity (Foucaud et al., 1997). In general, Mn^2+^, Mg^2+^, Ca^2+^, Fe^2+^, K^+^, and Na^+^ are essential ions required by LAB for nutrient transportation and enzymatic activity (von Wright and Axelsson, 2011).

### Antifungal Activity of Lactic Acid Bacteria and Their Metabolites

Among the LAB species, the antifungal activities of *Lactobacilli*, *Pediococcus*, and *Leuconostoc* have been the most studied (Crowley et al., 2013). *Lactiplantibacillus plantarum* (*Lp. plantarum*), *Lacticaseibacillus rhamnosus* (*Lac. rhamnosus*), *Limosilactobacillus reuteri* (*Lim. reuteri*), and *Levilactobacillus brevis* (*Lev. brevis*) display the antifungal activity against a large spectrum of spoilage fungi such as *Penicillium* and *Mucor* strains. An overview of the antifungal activity of LAB cultures against spoilage fungi in different media is listed in Supplementary Table 1.

**FIGURE 1:**
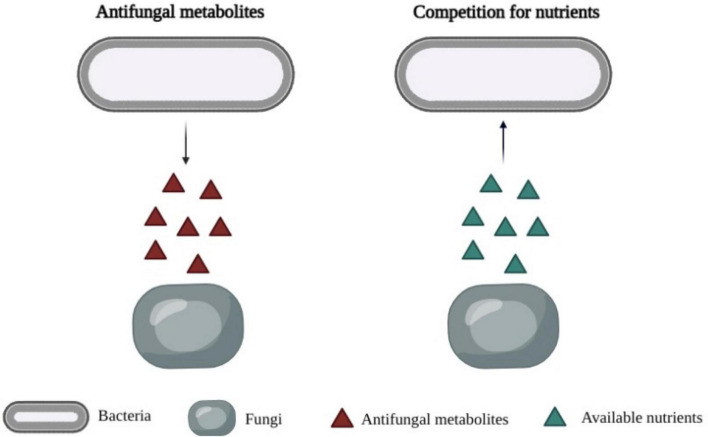
Primary mechanisms of antimicrobial activity of bioprotective cultures. Left: Production of antifungal metabolites by lactic acid bacteria. Right: Competition for available nutrients by lactic acid bacteria.

It was reported that LAB metabolites play a key role in the antifungal activity of LAB (Figure 1). Previous studies determined the antifungal effects of LAB metabolites in cell-free supernatants (CFS). Muhialdin et al. (2020) found that the cell-free supernatant of *Lp. plantarum* TE10 showed an inhibitory effect on the growth of *A. flavus*, and some peptides were characterized as antifungals. Russo et al. (2017) suggested that the growth of *P. expansum* CECT2278 was inhibited by the CFS of *Lp. plantarum* UFG108 and *Lp. plantarum* UFG121. The production of lactic acid and phenyllactic acid were found to be associated with fungal inhibition. Likewise, the antifungal properties of CFS of *Lp. plantarum*, *Limosilactobacillus fermentum* (*Lim. fermentum*) and *Latilactobacillus sakei* against *P. expansum* CECT2278 were determined by Yépez et al. (2017). The predominant bioactive compounds were identified as phenyllactic and 3,5-Di-O-caffeoylquinic acids.

The contribution of antifungal volatiles produced by LAB could not be overlooked or underestimated. Three volatile compounds in high amounts produced by LAB in sour cream (diacetyl, acetoin, and an unidentified volatile compound) displayed inhibitory effects on the growth of *P. commune* (Leyva Salas et al., 2019). Arrebola et al. (2010) also reported the antifungal activities of volatiles such as diacetyl, acetoin, 2-butanone, and volatile fatty acids. Aunsbjerg et al. (2015a) identified a main volatile, diacetyl, in the headspace of *Lacticaseibacillus paracasei* (*Lac. paracasei*) cell-containing fermentate in a defined medium, exhibited strong inhibitory effects on two *Penicillium* strains.

The interactions between the microbial metabolites produced by different bioprotective cultures might be involved in the antifungal effects. Lee et al. (2020) suggested that in LAB combinations, the LAB types and their inoculation ratios markedly influenced the produced metabolites types and concentrations during kimchi fermentation. Sahoo and Jayaraman (2019) found that a combination of *Lactobacillus delbrueckii* (*L*. *delbrueckii*) and *Lactobacillus delbrueckii* subsp. *lactis* (*L. lactis*) TSG3 enhanced stoichiometric yield of D-lactic acid from whey permeate (lactose); Leyva Salas et al. (2019) indicated that the concentration of diacetyl produced by the combination of *Lp. plantarum* and *Schleiferilactobacillus harbinensis* (*S. harbinensis*) was 3.7 mg/kg in yogurt, while it was 1.9 mg/kg in the control group, which indicated that the binary combination produced a higher amount of diacetyl. Similarly, Schwenninger et al. (2008) demonstrated that the combination of *Lac. paracasei* subsp. *paracasei* SM20 and *Propionibacterium jensenii* increased the production of acetic acid and propionic acid, but decreased the concentration of lactate. It was hypothesized that the lactate was consumed by the combination.

### Lactic Acid Bacteria Used as Bioprotective Cultures

Regarding the bioprotective cultures used in food products, in addition to being effective on the spoilage fungi, being safe for human consumption is more important. The selected bioprotective cultures should have no negative impact on food quality properties and human health, exhibit, and maintain active during food manufacturing and storage (Holzapfel et al., 1995). The main desirable requirements of bioprotective cultures in food products include: (1) The cultures should be GRAS; (2) Remain active at the lowest possible inoculum during product manufacturing and distribution to reduce the cost; (3) Being resistant to the antibiotic, lyophilization or freezing; (4) No production of any substances harmful to humans, such as biogenic amine; and (5) No effect on intrinsic sensory properties, such as flavor and texture.

From ancient times, LAB are widely used in food production due to their ability to produce efficient metabolites such as lactic acid and synthesize a wide range of metabolites that positively impact the nutritional, sensorial, and technological properties of food products (Pawlowska et al., 2012). The safety of LAB has been evaluated by different researchers to a limited extent in human beings. LAB have been granted the status of GRAS and QPS by the American Food and Drug Agency (FDA) and the European Food Safety Authority (EFSA), respectively. Thus, LAB have been extensively used as starter cultures of fermented food products, probiotics. Most importantly, LAB are considered as promising bioprotective cultures against unwanted organisms in food products, especially in fermented dairy products.

In recent years, researchers aim to screen out more efficient bioprotective LAB cultures. The antifungal activity of *Lac. paracasei* DGCC 2132 against *Penicillium* sp. nov. DCS 1541 and *P. solitum* DCS 302 in yogurt was assessed by Aunsbjerg et al. (2015b). In this study, the yogurt was produced with *Lac. paracasei* DGCC 2132 as the adjunct culture, and then the antifungal activity of LAB was determined by spotting the mold suspensions of the two *Penicillium* strains on the surface of the yogurt. The results revealed the antifungal properties of *Lac. paracasei* DGCC 2132 compared with the control samples after 4 days of treatment. Likewise, Cosentino et al. (2018) indicated that the selected *Lactobacilli* strains, including three *Lp. plantarum* strains and one *Lev. brevis* strain might have a potential effect in controlling the contamination caused by *P. chrysogenum* ATCC9179 and *A. niger* ATCC 46283 on cheeses without altering the sensory characteristics. Lynch et al. (2014) found that *Lactobacillus amylovorus* (*L. amylovorus*) DSM 19280 was an effective antifungal strain, showing inhibitory effects on a range of spoilage fungi, such as *P. expansum*, *P. roqueforti*, *A. niger*, *Aspergillus fumigatus* and *Fusarium culmorum* (*F. culmorum*), and thereby extend the shelf-life of Cheddar cheese. Comparing with the results in the control group, the presence of the antifungal *L. amylovorus* adjunct resulted in a 4-day delay in the growth of *Penicillium* on the cheese. In addition, synergistic interactions of LAB combinations might induce an increase in antifungal activity. Salas et al. (2018) suggested that the binary combination composed of *Lp. plantarum* L244 and *S. harbinensis* L172 delayed the growth of *P. commune*, *M. racemosus* and *Rhodotorula mucilaginosa* (*R. mucilaginosa*) on sour cream for 2–24 days without the effect on organoleptic properties at 10^6^ CFU/mL. Besides, the combination of *Lp. plantarum* L244 and *Lac. rhamnosus* CIRM-BIA1113 delayed the growth of *P. commune* in semi-hard cheese for 1 day. In our previous work (Shi and Knøchel, 2021), *Lp. plantarum* LP37 and *Lp. plantarum* LP48 exhibited positive interaction on the mold growth in yogurt serum agar plates. The antifungal effect of the combination significantly improved. In addition to the interactions between LAB cultures, the combinations of LAB with other bacterial strains such as propionic acid bacteria were also studied. Schwenninger and Meile (2004) suggested that the antifungal effect of *Lac. paracasei* subsp. *paracasei* strains (SM20, SM29, or SM63) increased due to the combination with *Propionibacterium jensenii* SM11. The combined cultures delayed the growth of yeasts in yogurt and cheeses at 6°C without affecting the quality properties of the dairy products. Besides, Fernandez et al. (2017) indicated that synergistic antifungal activity was observed between *Lac. rhamnosus* A238 and *Bifidobacterium animalis* against *P. chrysogenum* in cottage cheese. Currently, there are some bioprotective cultures available commercially to control the fungal spoilage of fermented dairy products. HOLDBAC^®^ YM-XPM (DuPont ApS, Denmark) composed of co-cultures of *Lac. paracasei* and *Lp. plantarum* are used in yogurt production to control the growth of spoilage yeasts and molds; HOLDBAC^®^ YM-XPK (*Lp. plantarum*) is applied as bioprotective culture in all types of cheeses; FreshQ^®^ (Chr. Hansen, Denmark) are composed of bioprotective cultures acting synergistically with the starter culture of fermented milk products against spoilage fungal organisms.

## Antifungal Metabolites Produced by Lactic Acid Bacteria

**FIGURE 2:**
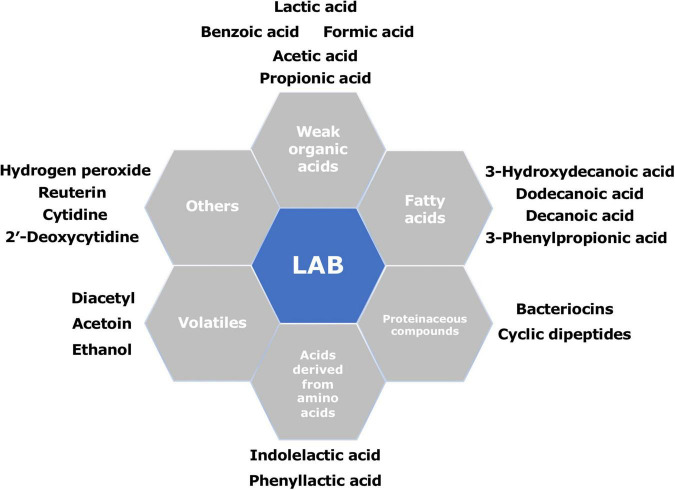
Summary of the main antifungal compounds produced by different lactic acid bacteria (LAB).

Previous studies have identified and characterized various antifungal metabolites produced by LAB (Figure 2). These metabolites range from simple primary metabolites to complex secondary metabolites derived from bioconversions or peptide synthesis and protein cleavage. The primary metabolites follow the growth of the microorganism, whereas the secondary metabolites are produced near the end of the growth phase (Madigan et al., 2008). The majority of identified antifungal substances are low-molecular-weight compounds, composed of organic acids, fatty acids, proteinaceous compounds, reuterin, hydrogen peroxide, and some volatiles. Supplementary Table 2 shows an overview of various metabolites produced by different LAB known to be involved in their antifungal performance.

### Weak Organic Acids

So far, weak organic acids are regarded as the major fermentation end products of carbohydrate metabolism of LAB, which include lactic acid, acetic acid, propionic acids, azelaic acid, hydrocinnamic acid, DL-phenyllactic acid, DL-hydroxyphenyllactic acid, *p*-coumaric acid, polyporic acid, 2-hydroxybenzoic acid, 4-hydroxybenzoic acid, vanillic acid, caffeic acid, succinic acid, 2-pyrrolidone-5-carboxylic acid, etc (Leyva Salas et al., 2017). Among the organic acids produced by LAB, the best characterized and most effective metabolites produced by LAB are lactic and acetic acid, which are bioactive in their protonated form at low pH (Arena et al., 2016). Garnier et al. (2020) also reported that lactic and acetic acids were the most abundant fermentation products for *Lac. rhamnosus*. However, the MICs of organic acids against spoilage fungi are often higher than the concentrations of organic acids produced by bioprotective cultures. Lactic acid produced by *Lp. plantarum* VE56 was around 77 mM, while the minimal inhibitory concentration (MIC) of lactic acid against *A. niger* CWBIF194 and *P. crustosum* MY1 was 1220 and 555 mM, respectively. Therefore, it is generally recognized that other antifungal metabolites are also involved in inhibiting unwanted organisms. For example, acetic acid is believed to have a synergistic effect with lactic acid in preventing fungal growth (Delavenne et al., 2013). The antifungal effect of *S. harbinensis* K.V9.3.1.Np to the synergistic action of lactic, acetic, 2-pyrrolidone-5-carboxylic, (S)-(-)-2-hydroxyisocapric, and 2-hydroxybenzoic acids was attributed by Mieszkin et al. (2017). Other organic acids derived from LAB are also receiving attention as antifungal agents. 3-Phenyllactic acid produced by *Lp. plantarum* was around 0.05 mg/mL. The addition of 3-phenyllactic acid prevented protein degradation during the ensiling period and improved the quality of alfalfa silage (Wu et al., 2020).

The antifungal mechanism of weak organic acids is hypothesized to act on the plasmic membrane. According to Freese et al. (1973), the electrochemical potential of the cell membrane is neutralized by interaction with acetic acid and propionic acid, then cell membrane permeability is increased afterward, which eventually leads to the death of susceptible organisms. To be specific, the weak organic acids are in a pH-dependent equilibrium between the undissociated and dissociated state in the solution. At low pH, organic acids show the optimal antimicrobial activity since low pH favors the molecule at uncharged and undissociated form, which makes it easier for the organic acids to permeable across the plasma membrane freely and finally penetrate the cell. Due to the higher pH inside the cell, the molecule will dissociate, leading to the release and accumulation of anions and protons, which cannot diffuse from the cells inside (Brul and Coote, 1999). In this context, the antimicrobial activity of organic acids is proposed to be due to the stress on the intracellular pH homeostasis, membrane disruption (Bracey et al., 1998), accumulation of toxic anions (Eklund, 1985), and inhibition of essential metabolic reactions (Krebs et al., 1983).

### Fatty Acids

Fatty acids are a class of organic acids characterized by the presence of a carboxyl-group (-COOH) on one end and a methyl chain (-CH3) on the other (Pohl et al., 2011). Some LAB can produce fatty acids such as 3-Phenylpropionic acid, decanoic acid, 3-hydroxydecanoic acid, and ricinoleic acid. 9,12-Otadecadienoic acid (Z,Z)-methyl ester, hexadecanoic acid, and methyl ester were identified as the main fatty acids found in n-hexane fractions of *Lp. plantarum* and *Loigolactobacillus coryniformis* subsp. *coryniformis* (*Loig. coryniformis*), respectively (Bukhari et al., 2020). The bacterial production of fatty acids is able to improve the sensory quality of fermented products, and some of them also possess antimicrobial activity. Three active antifungal compounds: 5-Oxododecanoic acid, 3-OH-decanoic acid, and 3-OH-5-dodecenoic acid, identified from *Lp. plantarum* HD1 by Ryu et al. (2014), showed inhibitory effect on a range of molds and yeasts. A novel fatty acid (12-hydroxydodecanoic acid) has also been found as a potential metabolite against filamentous fungi.

Fatty acids presented diverse antifungal activity depending on the length of the chain and the presence of unsaturated bonds. It was reported that antifungal activities of straight short and medium-chain fatty acids (C1 to C12) increase with increasing chain length (Woolford, 1975). More active was found to possess a 12-carbon atom chain length such as dodecanoic acid and lauric acid. Leyva Salas et al. (2019) found that *P. commune* inhibition in yogurt was associated with the higher amounts of four free fatty acids, including decanoic acid (C10:0), dodecanoic acid (C12:0), 9-Hexadecenoic acid (C16:1), and (9Z)-9-octadecenoic acid (C18:1). In general, saturated fatty acids were found to present higher antifungal activity than unsaturated acids (Pohl et al., 2011). Liu et al. (2008) found that palmitic acid, the saturated fatty acid, presented more potent inhibitory activity against fungi than oleic acid, an unsaturated fatty acid. However, short-chain and unsaturated fatty acids have presented remarkably stronger antifungal activity while lower concentrations of these fatty acids were detected. The inhibitory effect of polyunsaturated fatty acids was more effective than unsaturated long-chain fatty acids (Gołebiowski et al., 2014). For example, a polyunsaturated fatty acid, linoleic acid (18:2), has been reported to possess the inhibitory effect on several plant pathogenic fungi, whereas oleic acid failed to inhibit fungal growth (Liu et al., 2008). Besides, synergistic interaction among the bioactive compounds also contributes to antifungal activity. Three active components, including n-Decanoic acid (saturated fatty acid), 3-hydroxydecanoic acid, and 3-hydroxydodecanoic acid (hydroxy fatty acids), were detected in the CFS of *Lim. reuteri* by Sadeghi et al. (2019), and it was found the antifungal activity of the crude CFS was higher than the one of the antifungal fractions.

To date, although the knowledge about the antimicrobial mechanism of fatty acids is limited, the mode of action of *cis*-9-heptadecenoic acid, a fatty acid produced by *Pseudozyma flocculosa* against several plant pathogenic fungi, has been reported by Avis and Bélanger (2001). It was proposed that the lipid bilayers of fungal membranes are partitioned by the acids, leading to the increase of membrane fluidity and permeability, and ultimately resulting in cytoplasmic disintegration of fungal cells.

### Proteinaceous Compounds

The antifungal activity of some bioprotective cultures decreased with the addition of proteolytic enzymes such as trypsin, indicating proteinaceous compounds to be involved in the antifungal activity (Gerez et al., 2013). Proteinaceous compounds produced by LAB consist of ribosomal peptides such as bacteriocins, non-ribosomal peptides, and peptides derived from enzymatic hydrolysis of proteins. In comparison with the compounds described in previous studies, few researchers paid attention to the antifungal activity of proteinaceous compounds produced by LAB.

An example of proteinaceous compounds is bacteriocins, which are small, low-weight, cationic, ribosomally synthesized peptides with antimicrobial properties (Mora-Villalobos et al., 2020). However, bacteriocins are generally active against closely related bacteria, and thus, a number of researches reported the antibacterial activity of bacteriocins, whereas few literatures studied on the antifungal activity. Shehata et al. (2018) identified a new bacteriocin from *Lac. paracasei* KC39 which was isolated from a traditional Egyptian cheese (Kareish). The bacteriocin exhibited antifungal activity against toxigenic fungi growth, *Aspergillus parasiticus* (*A. parasiticus*) ITEM11, *Aspergillus carbonarius* ITEM 5010, and its excreted mycotoxin. Nevertheless, studies have reported that *Lactococcus*, *Streptococcus*, *Lactobacilli*, and *Pediococcus* were able to produce antifungal proteinaceous compounds to inhibit the growth of a broad spectrum of food-associated fungi. Among these genera, *Lactobacillus* are the most predominant isolates associated with such proteinaceous antifungal compounds (Crowley et al., 2013).

Apart from bacteriocins, antifungal peptides exhibit a broad inhibition spectrum against fungi, and thus have great potential to control spoilage fungi in fermented foods. Recently, a 9-amino acid antifungal peptide, pepa4c177, was identified by Garnier et al. (2020) in *Lac. rhamnosus* fermentates. The peptide showed an inhibitory effect on *M. racemosus* and *R. mucilaginosa* at the highest tested concentrations of 2.5 and 5 mg/mL, respectively, while *M. racemosus* could be inhibited at 1 mg/mL. Thirty-seven antifungal peptides with molecular weight ranged from 664 Da to 2024 Da were isolated from *Lp. plantarum* TE10 and identified by Muhialdin et al. (2020). A significant reduction of *A. parasiticus* (73%) and *P. expansum* (58%) growth was observed after 48 h using all the concentrations of SGADTTFLTK peptide, which was produced by *Lp. plantarum* (Luz et al., 2017). Similarly, the antifungal effect of peptide (AMPs LR14) produced by *Lp. plantarum* LR14 against *A. niger*, *Rhizopus stolonifera*, *M. racemosus*, and *P. chrysogenum* on spore germination and hyphal growth was investigated by Russo et al. (2017). Gerez et al. (2013) found a novel antifungal peptide was able to achieve 85% inhibition on *A. niger* CH101 respected to control. The peptide was smaller than 10 kDa, thermostable, active in the pH range of 4–7, and sensitive to trypsin.

The antifungal mode of action of peptides was speculated to be linked with cell wall damage. Muhialdin et al. (2020) identified 22 cationic peptides, which showed high antifungal activity against *A. flavus* by causing cell wall lysis and mycelia damage. McNair et al. (2018) identified a new antifungal peptide (DMPIQAFLLY; 1211 Da) produced by bioprotective *Lactobacilli* cultures in sour cream. The peptide significantly reduced the growth rate of *Debaryomyces hansenii* (*D. hansenii*) at concentrations ≥35 μM, and resulted in morphological changes of cells.

### Diacetyl

Diacetyl, an aromatic compound known as 2,3-butanedione (a polar, hydrophobic diketone with the relatively simple structure of CH3–CO–CO–CH3), is noted for its buttery aroma and taste. Diacetyl is found in many dairy products such as butter, yogurt, margarine, sour cream, and some cheeses (Hernandez-Valdes et al., 2020). In addition to dairy products, diacetyl is also found in roasted coffee, red and white wine, brandy, and other fermented food products (Jay, 1982). The levels of diacetyl measured in selected food are shown in Table 3 (Clark and Winter, 2015).

**TABLE 3:** The levels of diacetyl measured in selected foods (Clark and Winter, 2015).

Food	Range of diacetyl content in foods (ppm except where noted)
Butter	0.48–4.0
Cottage cheese	0.02–4.0
Cheddar cheese	0.23–0.76
Coffee	2.66–2.78
Goat milk Jack cheese	5.97–13.68
Margarine	0.48–27.0
Microwave popcorn	2–24 ppm; 0.64–0.92 mg emitted per bag
Wine	0.2–7.0
Yogurt	0.2–16.7

Moreover, diacetyl is used as a food additive in food products such as microwave popcorn due to its safety. Diacetyl has been granted GRAS status by the U.S. Food and Drug Administration (FDA) due to the safety of human exposure to diacetyl in fermented foods for centuries, Some LAB species, including *Streptococcus*, *Leuconostoc*, *Lactobacilli*, *Pediococcus*, and *Oenococcus*, can produce diacetyl. Aunsbjerg et al. (2015a) suggested that co-fermentation of the *Lac. paracasei* DGCC 2132 strain at 43°C with a yogurt starter culture markedly increased the production of diacetyl compared to yogurt without *Lac. paracasei* DGCC 2132; Lo et al. (2018) reported that twelve out of thirteen *Lac. rhamnosus* strains produced high diacetyl. The production of diacetyl by LAB could be affected by various factors, such as pH, substrates, and temperature. Aunsbjerg et al. (2015a) suggested that diacetyl production rapidly increased at pH below 5. Similarly, Branen and Keenan (1971) observed the production of diacetyl rapidly increased at pH below 5.5, with the highest production between pH 4.5 and 5.5. Jyoti et al. (2003) indicated that the productivity of diacetyl on a medium containing glucose and citrate doubly increased than that of a medium containing only citrate. Regarding the effect of temperature on diacetyl production, Bakirci et al. (2002) found that the amount of diacetyl produced at 4 ± 1°C was 0.8 ppm. However, it increased to 1.60 ppm at −18 ± 2°C, indicating a rapid equilibration in diacetyl levels at refrigeration temperatures.

In food products, diacetyl is not only the desirable flavor, but also an essential antimicrobial agent (Jay, 1982). The antifungal properties of diacetyl against several molds, including *Penicillium* spp. and *Fusarium* spp. were determined with the inhibition observed at concentrations above 86 μg/mL (Lagoni, 1941). Jay (1982) indicated the sensitivity of some molds and yeasts to diacetyl at the concentration of 100 and 200 μg/mL. Aunsbjerg et al. (2015a) found that the *Penicillium* strains were completely inhibited diacetyl with concentrations above 75 μg/mL for up to 5 days. Furthermore, the influence of the pH was strain-dependent. In our previous work, it was found that diacetyl inhibited the growth of *P. crustosum* 01180001 and *P. commune* 0118002 at the concentration of 64 μg/mL (Shi and Knøchel, 2021). In this research, we found that the underlying antifungal mechanism might be associated with membrane damage and oxidative stress. In addition, diacetyl possesses the capacity to inhibit the growth of both Gram-positive and Gram-negative bacteria, such as *Listeria, Salmonella, Escherichia coli (E. coli*), and *Aeromonas* (Ammor et al., 2006). The antibacterial mechanism was speculated to be related to the interference with the utilization of arginine in the periplasmic space (Ammor et al., 2006). Furthermore, Gram-negative bacteria, yeasts, and molds appear to be more sensitive toward diacetyl than Gram-positive bacteria (Jay, 1982).

### Hydrogen Peroxide

Production of hydrogen peroxide (H_2_O_2_) by LAB prevents the growth of spoilage organisms and foodborne pathogens. It plays an important role in food preservation since H_2_O_2_ is affirmed as GRAS for use in the food industry. According to previous work, a number of LAB are able to produce H_2_O_2_ in the presence of oxygen due to the existence of flavoprotein oxidase, NADH oxidases, and peroxidase enzymes (Gerez et al., 2013). Strains of lactobacilli, especially *L. delbrueckii* subsp. *lactis* produced more H_2_O_2_ in MRS broth at refrigeration temperatures (Jaroni and Brashears, 2000) and inhibited the growth of *E. coli* O157:H7 during refrigerated (7°C) storage (Brashears et al., 1998). Similarly, Yap and Gilliland (2000) reported that *L. delbrueckii* subsp. *lactis* produced a high amount of H_2_O_2_. Martín et al. (2005) characterized *Loig. coryniformis* as an H_2_O_2_-producing LAB from milk cheese. H_2_O_2_ has been used as an antimicrobial agent for a long time due to the efficiency to control or retard the growth of unwanted microorganisms in a variety of food products, including raw milk (Roundy, 1958), cantaloupes (Ukuku, 2004), and apples (Sapers and Sites, 2003).

Many researchers have reported the antimicrobial effect of H_2_O_2_. Venturini et al. (2002) confirmed the inhibitory effect of H_2_O_2_ at 5% and 10% on the growth of *P. expansum* on apple, and the MIC was described to be less than 0.025%. Ponts et al. (2006) suggested that the spore germination rate of *Fusarium graminearum* might be affected by H_2_O_2_. A significant reduction of mycotoxin accumulation induced by 0.5 mM H_2_O_2_ was also observed. The inhibitory effect of H_2_O_2_ is associated with the concentration, incubation time, and temperature (Roundy, 1958). For example, a concentration of 500 mg/L H_2_O_2_ for 30 min showed more effective antifungal activity on brown trout eggs than treatments at a concentration of 1000 mg/L (Novakov et al., 2018). Likewise, the addition of 0.0495% H_2_O_2_ induced the reduction of *Listeria monocytogenes* (*L. monocytogenes*) in sterilized milk but not at the concentration of 0.025% or 0.0125% (Dominguez et al., 1987). Temperature is also crucial in the antimicrobial activity of H_2_O_2_. Block (1991) found that H_2_O_2_ was weakly sporicidal at room temperatures but very potent at higher temperatures.

### Other Microbial Metabolites

Apart from the compounds produced by LAB described above, a range of other antifungal metabolites were found. Yang et al. (2011) described a novel antifungal compound, δ-dodecalactone, produced by *Lp. plantarum* AF1, which showed a strong inhibitory effect on molds, including *Aspergillus* strains and *P. roqueforti*.

Wang et al. (2012) reported the antifungal activity of benzeneacetic acid, 2-propenyl ester produced by *Lp. plantarum* IMAU10014 for the first time. *Penicillium citrinum* and *Penicillium drechsleri* were very sensitive to the compound.

Ryan et al. (2011) identified two antifungal nucleosides, cytidine, and 2′-deoxycytidine, in the cell-free supernatants of *L. amylovorus* DSM 19280. The MIC of the two compounds against *Aspergillus fumigatus* J9 was more than 200 mg/mL, which was much higher than other compounds in the supernatant. In addition, sodium decanoate was also identified to be antifungal, and the MIC was 0.05 mg/mL.

Reuterin (3-HPA, 3-hydroxypropionaldehyde) has been reported to possess the antimicrobial activity to inhibit a wide spectrum of Gram-positive and Gram-negative bacteria (Cleusix et al., 2007), yeast, molds and protozoa (Vimont et al., 2019; Greppi et al., 2020). The effect of reuterin produced by *Lim. reuteri* on the growth of *Helicobacter pylori* and virulence gene expression was evaluated recently (Urrutia-Baca et al., 2018). The growth was inhibited strongly by reuterin at concentrations ranging from 0.08 to 20.48 mM; MICs ranged from 10.24 to 20.48 mM. The MIC and minimal fungicidal concentration (MFC) of reuterin against filamentous molds and yeasts were determined by Vimont et al. (2019). The antimicrobial mechanism of reuterin was reported by Schaefer et al. (2010), who suggested that oxidative stress was generated in cells of *E. coli* due to the exposure to reuterin. The bioactive component of reuterin, the aldehyde group, interacts with thiol groups of small molecules and proteins, and causes oxidative stress to the cell, eventually leading to growth inhibition.

## Competitive Exclusion of Lactic Acid Bacteria

Competitive exclusion is a widespread phenomenon in nature, and it is considered as a mechanism to avoid the growth of unwanted microorganisms (Figure 1). It is defined as the microorganisms living together would compete for precisely the same resources (Winterhalder, 1980). The microbial competition includes the competition for nutrients, such as glucose, amino acids, the competition for a physical space, and the competition for essential ions, such as manganese (Siedler et al., 2020). Some examples of LAB as bioprotective cultures in the inhibition of unwanted organisms by competition nutrient or metal ions are shown in Table 4.

**TABLE 4:** Examples of LAB providing biocontrol against spoilage organisms through competition for nutrients or ions.

LAB	Target organism	Mechanism of inhibition	References
*Lac. paracasei*	*Penicillium solitum* DCS 302 *Penicillium sp. nov.* DCS 1541	Competition for glucose and glutamine	Honoré et al., 2016
*Lactobacillus hilgardii*	*Oenococcus oeni*	Competition for peptides	Fernández et al., 2010
*Lactococcus piscium* CNCM I-4031	*Listeria monocytogenes*	Competition for glucose, nitrogenous nutrients and vitamins, iron and magnesium	Saraoui et al., 2016
*Lactococcus piscium* CNCM I-4031	*Brochothrix thermosphacta*	Competition glucose, and amino-acids	Fall et al., 2012
*Lactococcus piscium* CNCM I-4031	*Listeria monocytogenes* RF191	Competition for nitrogenous nutrients and carbonic nutrients	Fall et al., 2010
*Lac. rhamnosus* R-2002	*Mucor plumbeus; Penicillium aurantioviolaceum*	Competition for glucose and glycerol	Toplaghaltsyan et al., 2017
*Lac. rhamnosus Lac. paracasei*	*Penicillium brevicompactum Penicillium crustosum Penicillium solitum Torulaspora delbrueckii Cryptococcus fragicola*	Competition for trace element, mainly for manganese ions	Siedler et al., 2020
*Lp. plantarum* LP37	*Penicillium crustosum Penicillium solitum Penicillium commune Penicillium glabrum Penicillium palitans Mucor plumbeus Mucor circinelloides*	Competition for trace element, mainly for manganese ions	Shi and Knøchel, 2021
*Lp. plantarum*	*Enterobacteriaceae*	Competition for trace element, mainly for manganese ions	Bruyneel et al., 1990
*Lp. plantarum* MDC9661	*Mucor plumbeus; Penicillium aurantioviolaceum*	Competition for trace element, mainly for magnesium ions	Matevosyan et al., 2020
*Lp. plantarum* MDC9661	*Mucor plumbeus; Penicillium aurantioviolaceum*	Competition for trace element, mainly for calcium ions	Matevosyan et al., 2020

*The names of LAB strains in the table are shown based on the new taxonomy (Zheng et al., 2020).*

### Competition for Essential Nutrients

The competition for nutrients indicates that LAB might inhibit the growth of spoilage fungi by consuming the available nutrients such as carbonic and nitrogenous nutrients, limiting or even depleting the pool of essential nutrients in the growth medium (Siedler et al., 2019). As indicated by Bayrock and Ingledew (2004), in addition to the microbial metabolites from LAB, competition for nutrients might be involved to inhibit the growth of yeasts.

According to Toplaghaltsyan et al. (2017), with the addition of a mixture of 10 g/L glucose and 10 mL/L glycerol, *Lac. rhamnosus* R-2002 lost the antifungal activity, the growth of *M. plumbeus* and *Penicillium aurantioviolaceum* (*P. aurantioviolaceum*) was observed. Mille-Lindblom et al. (2006) found that when bacteria were allowed to establish in the cultures before fungi were inoculated, the fungi did not grow at all. However, when the extra substrate was added along with the fungal inoculation, the fungi could overcome the total inhibition of growth, and reached biomass similar to that in cultures where fungi and bacteria were simultaneously inoculated. These studies suggest that competition for the substrate plays an important role in fungal inhibition. Likewise, Honoré et al. (2016) suggested that glucose and glutamine in a chemically defined medium were almost entirely consumed by *Lac. paracasei*. Other nutrients remained at 50% or more of the initial content. This is speculated to have the highest correlation with the inhibition of mold growth.

### Competition for Metal Ions With the Focus on Manganese

Apart from the competition for essential nutrients, the competition for metal ions was also reported by previous research, such as magnesium, calcium, and manganese. The role of competition for magnesium ions in the antimicrobial activity of LAB has become of great interest. Matevosyan et al. (2020) suggested that the presence of Mg^2+^ inhibited the antifungal property of *Lp. plantarum* against the growth of *M. plumbeus* and *P. aurantioviolaceum*. Calcium, an essential metal for LAB growth, can stimulate the proteolytic activity of some LAB (Keryan et al., 2017). The antifungal properties of LAB through the competition for calcium ions were reported by Matevosyan et al. (2020): *Lp. plantarum* MDC9661 lost the antifungal effects on the growth of *M. plumbeus* and *P. aurantioviolaceum* due to the addition of calcium ions. In this review, manganese depletion by LAB is mainly presented.

#### Response of Lactic Acid Bacteria to Manganese

**FIGURE 3:**
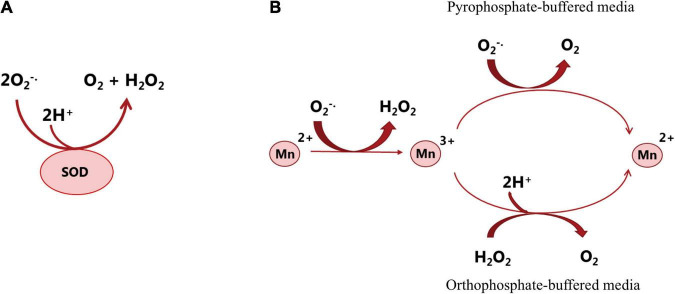
Dismutation of superoxide (O2^–^) by superoxide dismutase **(A)** or manganese **(B)**. SOD reacts with O_2_^–^ and generates H_2_O_2_ and O_2_ that are less reactive (Melo et al., 2011). Studying the catalytic activity of manganese in different *in vitro* systems (in pyrophosphate or orthophosphate-buffered media) showed that in both situations, Mn^2+^ is oxidized by O_2_^–^, which forms Mn^3+^ and H_2_O_2_ and then the formed Mn^3+^ is re-reduced to Mn^2+^, and O_2_ is produced. In the presence of pyrophosphate, O_2_^–^ acts as both oxidizing and reducing agent; in the presence of orthophosphate or some other buffered media, another reductant such as NAD(P)H or H_2_O_2_ is required for the scavenging activity of manganese. The above-mentioned mechanisms potentially occur in the bacterial cell. However, the reaction rate and the reaction course are determined by the ligands that are available and can associate with manganese (Archibald and Fridovich, 1981).

A number of microorganisms including LAB need at least trace amounts of Mn^2+^ for important metabolic functions, such as the production of secondary metabolites (Papp-Wallace and Maguire, 2006), the structure and activation of numerous enzymes, e.g., glutamine synthetase, RNA polymerase, lactate dehydrogenase, alkaline phosphatase (Terpstra et al., 2001), catalase, and superoxide dismutase (SOD) (Archibald and Duong, 1984). For the growth of LAB, manganese is not only an essential component in some enzymes, but also a cofactor in some metalloenzymes to assist cellular metabolism (Schmidt et al., 2016). However, the role of manganese is not limited to enzyme-mediated catalysis. The presence of manganese is crucial for the proper function of a variety of bacterial products, including the secretion of antibiotics and contribution to the stabilization of bacterial cell walls. In addition to the biological function of manganese as enzyme-mediated catalysis, manganese can detoxify reactive oxygen species (ROS) in cells lacking enzymatic defense such as SOD, catalases, and peroxidases. Most prokaryotes and all eukaryotes contain SOD to protect the cell from oxidative damage (Jakubovics and Jenkinson, 2001). However, LAB, even though being aerotolerant, lack an active SOD gene, except for lactococci and streptococci that are reported to have SOD activity. To cope with ROS, LAB accumulate high levels of intercellular manganese. For example, a high level of manganese ions is accumulated up to 25 mM by *Lp. plantarum* strains to scavenge superoxide for optimal growth due to the lack of SOD (Böhmer et al., 2013; Serata et al., 2018). During fermentative growth or in the presence of O_2_, manganese ions are used by LAB to scavenge the endogenous toxic oxygen species, particularly superoxide radical anion as effectively as SOD (Horsburgh et al., 2002). Figure 3 shows the dismutation of superoxide (O2^–^) by SOD or manganese. It is worth noting that the defense mechanism (accumulation of high level of manganese) has been found in some LAB species, such as *Lp. plantarum*, *Lacticaseibacillus casei* (*Lac. casei*) and *Lim. fermentum*. However, it is not present in all members of LAB, such as *L. delbrueckii subsp. bulgaricus* and *Lactobacillus acidophilus* (Archibald and Fridovich, 1981).

Manganese uptake by LAB strains occurs in an active manganese ion transport system. The genome analysis of *Lp. plantarum* by Groot et al. (2005) identified three types of manganese transporters, Mn^2+^/Cd^2+^ specific P-type ATPase (MntA), ATP-binding cassette (ABC) transporter (*mtsCBA*), and three proton-dependent natural resistance-associated macrophage protein (Nramp) transporters (*MntH1*, *MntH2*, and *MntH3*). The *mntA* gene in *Lp. plantarum* encodes for the high-affinity Mn^2+^/Cd^2+^ P-type ATPase transporter, where Cd^2+^ and Mn^2+^ compete for uptake in this system. The ABC transporter has three parts: an extra-cytoplasmic solute-binding protein, a cytoplasmic ATP-binding protein, and an integral membrane protein. These parts are encoded by an ABC transporter operon. ABC transporter is regulated through the operon, which is activated upon manganese starvation. Nramp family transporters are widely conserved and expand in all living organisms. The Nramp transporters facilitate the uptake of divalent metal ions by harnessing the proton gradient. Bacterial Nramp (*MntH*) that contributes to manganese uptake is a homolog of mammalian Nramp transporters for uptake of dietary iron (Groot et al., 2005). Nramp-type transporter (MntH) and ABC transporter (SitABC) are the major manganese uptake system in LAB. These transporters have likely distinct physiological roles in bacterial cells. The ABC transporters are mostly active in neutral or slightly alkaline pH, whereas acidic conditions activate the proton-derived *MntH* transporters (Porcheron et al., 2013; Siedler et al., 2020).

Transition metal ions such as manganese are required for a variety of biological processes. The unique redox potential of the transitional metals allows them to act as a cofactor for many enzymes. However, transitional metals in high concentrations are toxic due to the generation of highly reactive free radicals and disturbance of the redox potential of the cell (Porcheron et al., 2013). Therefore, manganese must be maintained at a steady-state balance in organisms; otherwise, stress can be resulted from manganese starvation or excess of manganese, leading to physiological function disruption (Hsieh et al., 2013). Manganese homeostasis is regulated by *MntR*, which is a sensor for manganese abundance. When *MntR* is bound to manganese, it binds with high affinity to the inverted repeat region in the promoter of the Mn transporter gene and represses the transcription of *sitABC* and *MntH*. In addition to repression of the manganese uptake, *MntR* activates the manganese efflux pump (*MntP*) (Horsburgh et al., 2002; Porcheron et al., 2013). Tong et al. (2016) reported that the Mn regulator *MntR* and transporter *MntH1-3* in *Lp. plantarum* CCFM436 were the major functional elements in Mn transportation regulation. *MntH1*, *MntH2*, and *MntH3* were down-regulated by *MntR*. However, in fact, manganese homeostasis in bacteria is regulated in a complex process that overlaps with iron homeostasis and peroxide defense since the cell must simultaneously regulate the demanding concentration of manganese and iron in response to the level of ROS (Horsburgh et al., 2002; Porcheron et al., 2013). In prokaryotes, manganese transport is regulated at the transcriptional level; while in eukaryotes; the manganese regulation is post-transcriptional. The concentration of intercellular manganese is regulated by targeted production or degradation of manganese transporters in response to changes in extracellular manganese concentrations (Costa and Aschner, 2015).

#### Effect of Manganese on the Bioprotective Mechanism of Lactic Acid Bacteria

Recently, Siedler et al. (2020) suggested that competitive exclusion through manganese depletion is a major mechanism employed by LAB to inhibit the growth of dairy spoilage yeast and molds in fermented milk. which naturally contains a trace amount of manganese. They reported that the inhibition or reduction of spoilage yeast and mold growth is due to the uptake of the trace amount of manganese in dairy products by bioprotective cultures. Therefore, the inhibition of mold growth in yogurt and the capability of manganese to restore mold growth appears to be associated with the ability of LAB to efficiently scavenge manganese. Exploring the inhibitive effect of a *Lp. plantarum* (Shi and Knøchel, 2021), it was found that the addition of increasing manganese concentrations (up to 0.1 mM) correlated positively with the growth of the five sensitive *Penicillium* (*P. crustosum, P. commune, P. solitum, P. glabrum*, and *P. palitans*) and two *Mucor* strains (*M. circinelloides* and *M. plumbeus*), showing that manganese depletion played a dominating role in the inhibition observed. Studying the expression of genes involved in manganese transport can provide an insight into the role of manganese depletion by bioprotective bacterial cultures. As mentioned above, the expression level of manganese transporters is regulated in response to the variations in the manganese concentration to maintain the manganese homeostasis, which might be regulated by multiple genes (Groot et al., 2005; Serata et al., 2018). Siedler et al. (2020) observed that the manganese transporter *MntH1* was the most expressed genes in both *Lac. rhamnosus* and *Lac. paracasei* strains in milk. Moreover, the deletion of *MntH1* gene in *Lac. paracasei* strains resulted in a loss of bioprotective activity despite the presence of *MntH1* and *MntH2* transporters and ABC transport system, indicating a correlation between the presence of *MntH1* gene and bioprotective activity in selected lactobacilli in fermented dairy products.

## Conclusion, Future Challenges, and Perspective

In recent years, the demand for “clean label” food products from consumers has been increasing. LAB and their bioactive metabolites are considered the natural chemical preservative replacement for use as the bioprotective tools in fermented dairy products to control the fungal spoilage and satisfy the consumer demands and legislation. The production of antifungal metabolites and competitive exclusion might be the main mechanisms of LAB for fungal growth inhibition. However, although numbers of studies have reported the antifungal efficiency of LAB cultures and/or bioactive metabolites in food products, only few LAB cultures are commercially used up to now. It is likely that some constraints may hinder the commercialization of the candidate antifungal LAB cultures, not all the criteria of bioprotective cultures in food products have been thoroughly satisfied. The first constraint concerns the safety issues. The safety assessment and risk analysis of the selected LAB cultures or their antifungal metabolites used in food preservation are the key points linked to the antifungal efficiencies and product quality issues. Therefore, it is crucial to take multiple criteria into account, and follow the rigorous safety assessment procedures. The second constraint seems to be the gap between an efficient antifungal activity in laboratory media (*in vitro*) and the actual antifungal activity in a real food matrix (*in situ*). Thus, it is important and necessary to carry out a challenge test to evaluate the actual antifungal activity of the potential LAB bioprotective cultures in the real food products.

As previously stated, a variety of antifungal compounds can be produced by LAB cultures, which is regarded as the main antifungal mechanism. According to previous research, a number of bioactive compounds have been identified, which are likely to act synergistic or additive bioactivity. In this context, the identification of new antimicrobial compounds, which are in low amounts, is hampered in complicated food matrices, such as yogurt. In addition, a number of low-molecular-mass compounds with antimicrobial activity might remain unidentified because of the lack of efficient technology. Thus, development of new sensitive detection approaches for low-molecular-mass compounds identification in food products should be considered. Furthermore, the combinations of LAB cultures or antifungal compounds should be further investigated, understanding the synergistic or additive effects. These combinations could be employed as novel biopreservatives to extend food shelf life.

From a consumer perception and legal viewpoint, the use of competitive cultures is non-controversial since no antimicrobials are needed. However, to what extent competitive exclusion by LAB plays a role has not been studied in depth. The role of competitive exclusion on the overall antifungal mechanism of LAB in various complicated food matrices remains to be further elucidated. Furthermore, based on our previous research (Shi and Knøchel, 2021), we found that the sensitivity toward antifungal metabolites and competition mechanism may differ among molds since the growth of *P. roqueforti* was not affected by manganese. Therefore, the molds that are sensitive to antifungal metabolites should be summarized as well as that are sensitive to manganese depletion. In addition to the antifungal metabolites produced by LAB cultures and competitive exclusion, the relationship between the physiology of the spoilage fungi and antifungal mechanism behind LAB cultures or bioactive compounds is required to be understood.

Overall, LAB cultures and their bioactive metabolites are promising candidates as the natural alternative preservatives, which not only reduce food spoilage and waste, satisfy the demand for “clean label” products from consumers, but also conserve economic and environmental resources, leading to a sustainable food industry especially dairy products for the future.

## Author Contributions

CS designed, wrote, and revised the manuscript. MM contributed to writing of the manuscript. Both authors contributed to the article and approved the submitted version.

## Conflict of Interest

The authors declare that the research was conducted in the absence of any commercial or financial relationships that could be construed as a potential conflict of interest.

## Publisher’s Note

All claims expressed in this article are solely those of the authors and do not necessarily represent those of their affiliated organizations, or those of the publisher, the editors and the reviewers. Any product that may be evaluated in this article, or claim that may be made by its manufacturer, is not guaranteed or endorsed by the publisher.
