# Supplementary material for: Lactic Acid Bacteria as Biopreservation Against Spoilage Molds in Dairy Products – A Review
Source: Front Microbiol. 2022 Jan 26;12:819684. doi: 10.3389/fmicb.2021.819684 (PMC8826399; doi:10.3389/fmicb.2021.819684)
Supplement: Supplementary file 1 [file Table_1.docx]

| **Table S1.** Active LAB cultures against fungal contaminants of food products. | | | | | | |
| --- | --- | --- | --- | --- | --- | --- |
| **Antifungal organisms** | **Source of antifungal organisms** | **Activity spectrum** | **Method of application** | **Antifungal compound(s)** | **Matrix** | **Reference** |
| ***Lactobacillus species*** |  |  |  |  |  |  |
| *Lac. rhamnosus  Lp. plantarum  Lev. brevis Lactiplantibacillus pentosus* (*Lac. pentosus*) *Lac. paracasei subsp. paracasei* | Camel’s milk | *Penicillium sp.  Aspergillus fumigatus* | Cell culture | Proteinaceous antifungal substances | MRS | (Eddine et al., 2018) |
| *Lp. plantarum* TE10 | Fermented soybeans | *Aspergillus flavus* MD3 | Cell-free Supernatant | Peptide | Maize seeds | (Muhialdin et al., 2020) |
| *Lp. plantarum* LB-1 | Pickles | *Penicillium citrinum Aspergillus niger Aspergillus flavus Aspergillus ochraceu Aspergillus fumigatus Fusarium graminearum* | Cell culture | Not evaluated | Wheat bread | ( Sun et al., 2020) |
| *Lim. reuteri* | Whole wheat sourdough | *Aspergillus niger* | Cell culture | n-Decanoic acid, 3-hydroxydecanoic acid  and 3-hydroxydodecanoic acid | Bread | (Sadeghi et al., 2019) |
| *Lim. fermentum* | Cocoa bean | *Aspergillus flavus* S07 | Cell culture | Not evaluated | Cocoa beans | (Romanens et al., 2019) |
| *Lac. paracasei* B20 and B6 *Lp. plantarum* CH1 | Raw milk | *Penicillium commune* | Cell culture | Organic acids | Sour cream | (Ouiddir et al., 2019) |
| *Lp. plantarum* CCDM181 | Silage | *Penicillium expansum* DMF04 *Fusarium culmorum* DMF301 | Cell culture and  Heat-treated bacterial supernatants | Organic acids | MRS | (Horackova et al., 2018) |
| *Lac. pentosus* LAP1 | Fermented fish product | *Candida tropicalis Candida albicans Candida krusei* | Cell-free Supernatant | Not evaluated | MRS | (Aarti et al., 2018) |
| *Lac. rhamnosus* MDC 9661 | Armenian dairy products | *Penicillium aurantioviolaceum  Mucor plumbeus* | Cell culture | Proteinaceous antifungal substances | MRS | (Bazukyan et al., 2018) |
| *Lac. pentosus* ŁOCK0979 | Culture collection center | *Aspergillus alternata Aspergillus ochraceus  Penicillium sp.* | Cell culture | Organic acids and fatty acids | MRS | (Lipińska et al., 2018) |
| *Lp. plantarum* ALAC-4 | Dairy products | *Candida albicans  Rhodotorula sp Aspergillus niger Penicillium sp Mucor hiemalis* | Cell culture | Proteinaceous antifungal substances | MRS | (Chen et al., 2018) |
| *Lp. plantarum* UFG121 | Food matrices | *Fusarium culmorum  Penicillium chrysogenum  Penicillium expansum Penicillium roqueforti Aspergillus flavus* | Cell culture and Cell-free supernatant | Organic acids | Oat-based product | (Russo et al., 2017) |
| *Lp. plantarum* M5MA1 | Fermented Andean products | *Penicillium roqueforti Aspergillus oryzae Meyerozyma guilliermondii* | Cell culture and Cell-free supernatant | Phenyllactic acid 3-Propanoic acid 2-Deoxycytidine Quercetinpentoside Quinic acid derivative 3,5-Di-O-caffeoylquinic acid | MRS | (Yépez et al., 2017) |
| *Lactobacillus helveticus* KLDS 1.8701 | Dairy products | *Penicillium spp.* | Cell culture | Organic acids | Fermented soybean milk | (Bian et al., 2016) |
| *Lp. plantarum* K46 | Fermented sesame leaf | *Gibberella moniliformis Aspergillus fumigatus* | Cell culture | Not evaluated | MRS | (Arasu et al., 2014) |
| *S. harbinensis* KV9.3.1Np | Cow and goat milk | *Debaryomyces hansenii,  Penicillium brevicompactum, Rhodotorula mucilaginosa,  Penicillium expansum* | Cell culture | Acetic and lactic acid | Milk and yoghurt | (Delavenne et al., 2013) |
| *Lp. plantarum* 16 | Steep water | *Rhodotorula mucilaginosa* | Cell culture and  Cell-free Supernatant | Not evaluated | Orange juice and yoghurt | (Crowley et al., 2012) |
| *Lev. brevis* NCDC02 | Culture collection center | *Candida guillermondii* (NCDC44) *Aspergillus niger* (NCDC 267) *Penicillium roqueforti* (NCDC170) *Penicillium camemberti* (NCDC56) *Rhizopus oryzae* (NCDC52) | Cell culture | Proteinaceous antifungal substances | MRS | (Falguni et al., 2010) |
| *Lim. reuteri* CRL1100 | Dairy products | *Fusarium graminearum* CH103 | Cell-free Supernatant | Acetic and phenyllactic acids | Bread | (Gerez et al., 2009) |
| *Lp. plantarum  Lac. pentosus* | Salami | *Aspergillus candidus* DSM814T *Penicillium nalgiovense* MFBP3 | Cell culture | Organic acids and peptidic compounds | MRS | (Coloretti et al., 2007) |
| ***Pediococcus species*** |  |  |  |  |  |  |
| *Pediococcus pentosaceus* | Wheat sourdough | *Aspergillus niger* | Cell culture | Fatty acid ester hydroxylated fatty acid ester cyclic dipeptide | Bread | (Ebrahimi et al., 2020) |
| *Pediococcus acidilactici* JY03 | Orange fruit | *Penicillium digitatum* | Cell culture and  Cell-free Supernatant | Proteinaceous compounds | Citrus | (Ma et al., 2019) |
| *Pediococcus acidilactici* No. 29 | Spontaneous sourdough | *Aspergillus nidulans  Penicillium funiculosum Fusarium poae Alternaria alternata* | Cell culture | Organic acids | MRS | (Bartkiene et al., 2020) |
| *Pediococcus pentosaceus Pediococcus acidilactici* | Spontaneous sourdough | *Aspergillus nidulans Penicillium funiculosum Fusarium poae* | Cell culture | Organic acids | MRS | (Bartkiene et al., 2020) |
| *Pediococcus acidilactici* HW01 | Malt | *Candida albicans* | Cell-free supernatant | Not evaluated | MRS | (Kim and Kang, 2019) |
| *Pediococcus acidilactici* | Acorn sourdough | *Aspergillus flavus* | Cell culture | Isopentyloxyethyl acetate Acetic acid Dihydro-4,4-dimethyl-2(3H)-furano  Decane Tridecane | Wheat bread | (Purabdolah et al., 2020) |
| *Pediococcus acidilactici* CRL1753 | Silage | *Aspergillus niger Aspergillus japonicus Penicillium roqueforti Metschnikowia pulcherrima* | Cell culture | Phenyllactic acid | Bread | (Bustos et al., 2018) |
| ***Leuconostoc species*** |  |  |  |  |  |  |
| *Leuconostoc mesenteroides* L1 | Raw milk | *Aspergillus tubingensis Aspergillus flavus* | Cell culture | Organic acids | Bread | (Ouiddir et al., 2019) |
| *Leuconostoc mesenteroides* No. 225 | Spontaneous sourdough | *Aspergillus nidulans Penicillium oxalicum Penicillium funiculosum Fusarium poae* | Cell culture | Organic acids | MRS | (Bartkiene et al., 2020) |
| *Leuconostoc fallax* L91 | Fermented wheat (lemzeiet) | *Rhodotorula mucilaginosa,  Aspergillus flavus Penicillium verrucosum* | Cell culture | Not evaluated | Wheat flour hydrolysate | (Merabti et al., 2019) |
| *Leuconostoc citreum, Leuconostoc mes. Dextranicm* | Culture collection center | *Penicillium commune Mucor racemosus* | Cell culture | Not evaluated | Cheese | (Salas et al., 2018) |
| *Leuconostoc citreum* | Sourdough | *Aspergillus niger  Penicillium chrysogenum* | Cell culture | Not evaluated | Bread | (Ispirli et al., 2018) |
| *Leuconostoc mesenteroides* T1M3 | Traditional fermented Andean products | *Meyerozyma guilliermondii Penicillium roqueforti, Aspergillus oryzae Aspergillus niger* | Cell cultures and  Cell-free supernatant | Not evaluated | MRS | (Yépez et al., 2017) |
| *Leuconostoc mesenteroides* TA | Kimchi | *Penicillium roqueforti* ATCC 10110 *Aspergillus spp.* | Cell-free supernatant | Lactic acid, acetic acid and  unidentified hydrophobic compound(s) | MRS | (Lee and Chang, 2016) |

The names of LAB strains in the table are shown based on the new taxonomy (Zheng et al., 2020).

| **Table S2.** List of compounds derived from LAB metabolic activity and their antimicrobial spectrum. | | | | | | | |
| --- | --- | --- | --- | --- | --- | --- | --- |
| **Compound** | **Producing Microorganisms** | **Source of Microorganisms** | **Production level** | **Matrix** | **MIC** | **Targeted Organisms** | **Reference** |
| Lactic acid | *Lac. rhamnosus* CIRM-BIA1952 | Culture collection center | 86.018 ± 3.05 mg/g | Fermented dairy media | N.D. | N.D. | (Garnier et al., 2020) |
|  | *Lp. plantarum* BCH-1 | Rice rinsed water | 773.7 μg/mL | MRS | N.D. | *Aspergillus favus  Aspergillus fumigatus* | (Bukhari et al., 2020) |
|  | *S. harbinensis* KV9.3.1Np | Cow milk | 235.86 ± 1.39 mM | Fermented milk supernatant | N.D. | *Yarrowia lipolytica* | (Mieszkin et al., 2017) |
|  | *Lp. plantarum Weissella cibaria  Weisella paramesenteroides* | Mill flour and fermented cassava | 44.8-76.8 mM | MRS | 555-1220 mM | *Aspergillus niger* | (Ndagano et al., 2011) |
|  | *Lp. plantarum* CH1 | Raw milk | 12.1 g/kg | Sourdough | N.D. | *Aspergillus tubingensis  Aspergillus favus* | (Ouiddir et al., 2019) |
| Acetic acid | *Lp. plantarum* M1 | Pickles | 11.28 ± 0.08 (mg/mL) | MRS | N.D. | N.D. | (Wu et al., 2020) |
|  | *Lactobacillus sp.* | Maize bran | 12.6 ± 3.4 - 16.2 ± 2.3 mM | Sourdough | 25.0 ± 5.5 (mM) | *Penicillium roqueforti FUA5005* | (Quattrini et al., 2019) |
|  | *Lactobacillus sp.* | Maize bran | 12.6 ± 3.4 - 16.2 ± 2.3 mM | Sourdough | 8.2 ± 3.4 (mM) | *Aspergillus niger* FUA5001 | (Quattrini et al., 2019) |
|  | *Lactobacillus mesenteroides* L1 | Raw milk | 0.64 ± 0.17 g/kg | Sourdough | N.D. | *Aspergillus tubingensis  Aspergillus favus* | (Ouiddir et al., 2019) |
|  | *Lactobacillus mesenteroides* L2 | Raw milk | 0.64 ± 0.3 g/kg | Sour cream | N.D. | *Aspergillus tubingensis  Aspergillus favus* | (Ouiddir et al., 2019) |
| Citric acid | *Lp. plantarum* BCH-1 | Rice rinsed water | 737.1 μg/mL | MRS | N.D. | *Aspergillus favus Aspergillus fumigatus* | (Bukhari et al., 2020) |
| Sorbic acid | *Lactobacillus sp.* | Maize bran | N.D. | Sourdough | 0.4 ± 0.1 (mM) | *Aspergillus niger* FUA5001 | (Quattrini et al., 2019) |
|  | *Lactobacillus sp.* | Maize bran | N.D. | Sourdough | 0.2 ± 0.0 (mM) | *Penicillium roqueforti* | (Quattrini et al., 2019) |
| Formic acid | *Fructilactobacillus sanfranciscensis* | Sourdough | 1.43 mM | Wheat flour hydrolysate | 19.50 mM | *Fusarium graminearum* | (Corsetti et al., 1998) |
| Succinic acid | *Lac. paracasei* B6 | Raw milk | 0.14 ± 0.01 (g/kg) | Sourdough | N.D. | *Aspergillus tubingensis  Aspergillus favus* | (Ouiddir et al., 2019) |
|  | *Lactobacillus sp.* | Culture collection center | 0.02-0.4 g/kg | Cheese, yoghurt, sour cream, semi-hard cheese | N.D. | *Penicillium commune* | (Leyva Salas et al., 2019) |
|  | *Lp. plantarum* CH0 | Raw milk | 0.21 ± 0.03 g/kg | Bread | N.D. | *Aspergillus tubingensis  Aspergillus favus* | (Ouiddir et al., 2019) |
|  | *Lp. plantarum* CH1 | Raw milk | 0.06 ± 0.01 g/kg | Fresh cream | N.D. | *Penicillium commune* | (Ouiddir et al., 2019) |
| Benzoic acid | *Lp. plantarm* | Culture collection center | 0.001 mg/g | MRS | 0.1-1 mg/mL | *Penicillium anomala Aspergillus fumigatus Penicillium roqueforti* | (Broberg, et al. 2007) |
| Azelaic acid | *Lactobacillus spicheri* O15 | Culture collection center | 2.71 ± 0.22 (mg/L) | MRS | N.D. | *Penicillium anomala Aspergillus fumigatus Penicillium roqueforti* | (Le Lay et al., 2016) |
| Indolelactic acid | *Lac. paracasei* B | DuPont Nutrition Biosciences ApS | 4.4 ± 0.2 mg/L | Defined media | 5 g/L | *Penicillium solitum* DCS 302 *Penicillium sp. nov.* DCS 1541 | (Honoré et al., 2016) |
| Propionic acid | *Lactobacillus sp.* | Maize bran | N.D. | Sourdough | 12.0 ± 0.0 (mM) | *Penicillium roqueforti* FUA5005 | (Quattrini et al., 2019) |
|  | *Lactobacillus sp.* | Maize bran | N.D. | Sourdough | 1.3 ± 0.2 (mM) | *Aspergillus niger* FUA5001 | (Quattrini et al., 2019) |
| 3-Phenyllactic acid | *Lp. plantarum* M1 | Pickles | 0.066 ± 0.000 (mg/mL) | MRS | N.D. | N.D. | (Wu et al., 2020) |
|  | *Pediococcus pentosaceus* SK25 | Traditional Chinese pickles | 0.81 ± 0.06 (mM) | MRS | N.D. | N.D. | (Yu et al., 2015) |
|  | *Lp. plantarum* CECT-221 | Spanish Collection of Type Cultures | 1.38 ± 0.048 (mM) | MRS | N.D. | *Salmonella enterica* | (Rodríguez et al., 2012) |
|  | *Lac. pentosus* | Spanish Collection of Type Cultures | 0.82 ± 0.009 (mM) | MRS | N.D. | *Salmonella enterica* | (Rodríguez et al., 2012) |
|  | *Lactobacillus acidophilus* | Spanish Collection of Type Cultures | 0.39 ± 0.04 (mM) | MRS | N.D. | *Salmonella enterica* | (Rodríguez et al., 2012) |
|  | *Lactobacillus sp.* | Maize bran | N.D. | Sourdough | 50 ± 0.00 (mM) | *Penicillium roqueforti* FUA5005 | (Quattrini et al., 2018) |
|  | *Lactobacillus sp.* | Maize bran | N.D. | Sourdough | 30 ± 10 (mM) | *Aspergillus niger*FUA5001 | (Quattrini et al., 2018) |
|  | *Lp. plantarum* VE56 | Fermented cassava | 0.48 ± 0.02 (mM) | MRS | 180 mM | *Aspergillus niger Aspergillus tubingensis Penicillium crustosum* | (Ndagano et al., 2011) |
|  | *Lp. plantarum* | Grass silage | N.D. | MRS | 6.5 - 12.0 mg/mL | *Penicillium sp. Aspergillus sp.* | (Prema et al., 2010) |
| Ricinoleic acid | *Lactobacillus sp.* | Maize bran | N.D. | Sourdough | 1.7 ± 0.0 (mM) | *Aspergillus niger*FUA5001 | (Quattrini et al., 2019) |
|  | *Lactobacillus sp.* | Maize bran | N.D. | Sourdough | 3.5 ± 0.0 (mM) | *Penicillium roqueforti* FUA5005 | (Quattrini et al., 2019) |
| Caproic acid | *Fructilactobacillus sanfranciscensis* | Sourdough | 0.88 mM | Wheat flour hydrolysate | 4.30 mM | *Fusarium graminearum* | (Corsetti et al., 1998) |
| Octadecanoic acid | *Lp. plantarum* BCH-1 | Rice rinsed water | 298.51 μg/mL | MRS | N.D. | *Aspergillus favus Aspergillus fumigatus* | (Bukhari et al., 2020) |
| Decanoic acid | *Lp.plantarum* CH1 | Raw milk | 1.23 ± 0.24 (mg/kg) | Sourdough | N.D. | *Aspergillus flavus  Aspergillus tubingensis* | (Ouiddir et al., 2019) |
| 2-Hydroxy-4-methyl- pentanoic acid | *Lp.plantarum* | Culture collection center | 0.001-0.0015 mg/g | MRS | 10 mg/mL | *Penicillium anomala Aspergillus fumigatus Penicillium roqueforti* | (Broberg et al., 2007) |
| 3-hydroxydecanoic acid | *Lp. plantarum* | Culture collection center | 0.036-0.21 μg/g | MRS | 0.1 mg/mL | *Penicillium anomala Aspergillus fumigatus Penicillium roqueforti* | (Broberg et al., 2007) |
| 2-Hydroxy-(4-methylthio) butanoic acid | *Lac. paracasei* | DuPont Nutrition Biosciences ApS | 0.2 ± 0.03 - 4.4 ± 0.2 (mg/L) | Defined media | 10 g/L | *Penicillium solitum* DCS 302 *Penicillium sp. nov.* DCS 1542 | (Honoré et al., 2016) |
| 2-Hydroxy-3-methylbutanoic acid | *Lac. paracasei* | DuPont Nutrition Biosciences ApS | 5.3 ± 0.03 - 23.4 ± 2.2 (mg/L) | Defined media | 5 g/L | *Penicillium solitum* DCS 302 *Penicillium sp. nov.* DCS 1543 | (Honoré et al., 2016) |
| 2-Hydroxy-4-methypentanoic acid | *Lac. paracasei* | DuPont Nutrition Biosciences ApS | 12.3 ± 0.2 - 65 ± 6 (mg/L) | Defined media | 6 g/L | *Penicillium solitum* DCS 302 *Penicillium sp. nov.* DCS 1544 | (Honoré et al., 2016) |
| Bacteriocin | *Lac. paracasei* KC39 | Traditional Egyptian cheese (Kareish) | N.D. | MRS | N.D. | *Aspergillus parasiticus ITEM11 Aspergillus carbonarius* ITEM 5010 | (Shehata et al., 2018) |
| Peptide pepa4c177 | *Lac. rhamnosus* | Dairy products | N.D. | MRS | 1-5 mg/mL | *Mucor racemosus Rhodotorula mucilaginosa* | (Garnier et al., 2020) |
| Peptide | *Lp. plantarum* TE10 | Fermented soybeans (tempeh) | N.D. | MRS | N.D. | *Aspergillus flavus* | (Muhialdin et al., 2020) |
| Hydrogen peroxide | *Leuconostoc citreum* L123 | Culture collection center | 0.58 ± 0.00 (μM) | MRS | N.D. | *Aspergillus niger Penicillium corylophilum* | (Le Lay et al., 2016) |
| Reuterin | *Lim. reuteri* ATCC 53608 | Swine intestine | N.D. | MRS | 0.2-2.0 mM | *Penicillium chrysogenum  Penicillium citrinum Penicillium commune Penicillium crustosum Penicillium roqueforti* | (Vimont et al., 2019) |
|  | *Lac. reuteri* | Chicken Gastrointestinal Tract | 156.9-330.2 mM | Glycerol solution | 0.02-3 μg/mL | *Penicillium spp.* | (Greppi et al., 2020) |
|  | *Lim. reuteri* 1063 | Culture collection center | N.D. | Basel medium | 0.15-1.12 mM | *Candida albicans Candida glabrata Saccharomyces cerevisiae Saccharomycopsis fibuligera* | (Chung et al., 1989) |
|  | *Lim. reuteri* 1064 | Culture collection center | N.D. | Basel medium | 0.56-3 mM | *Fusarium samfucienum Aspergillus flavus* | (Chung et al., 1989) |
| Diacetyl | *Lactobacillus sp.* | Culture collection center | 3.3-13.4 mg/kg | Cheese, yoghurt, sour cream, semi-hard cheese | N.D. | *Penicillium commune Mucor racemosus* | (Leyva Salas et al., 2019) |
|  | *Lac. paracasei* DGCC 2132 | Dairy product | 74 μg/g | Defined media | N.D. | *Penicillium solitum* DCS 302 *Penicillium sp. nov.* DCS 1544 | (Aunsbjerget al., 2015b) |

N.D. indicates no detection. The names of LAB strains in the table are shown based on the new taxonomy (Zheng et al., 2020).
